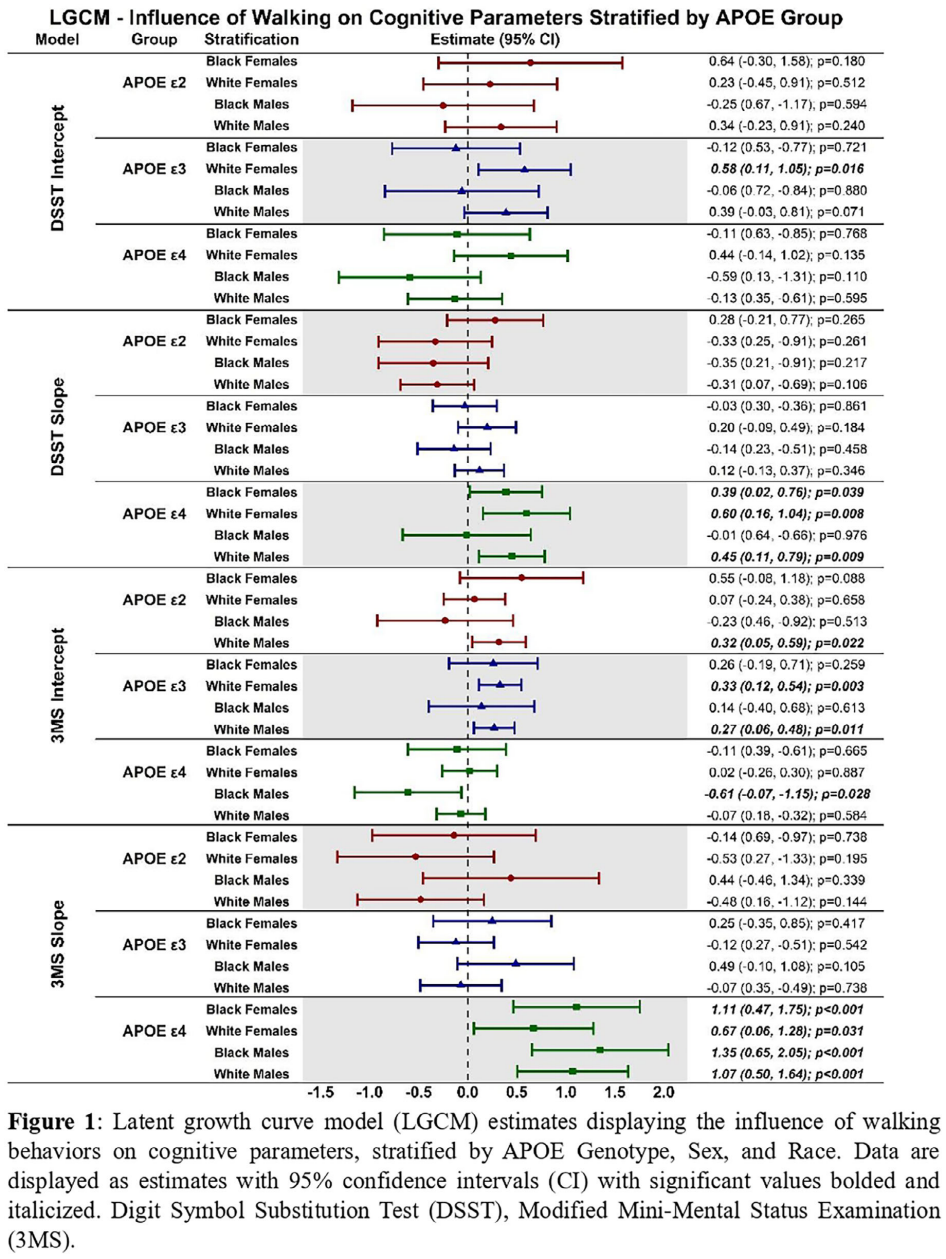# Walking to Protect Against Cognitive Decline: The Role of APOE Genotype, Sex, and Race

**DOI:** 10.1002/alz70860_103779

**Published:** 2025-12-23

**Authors:** Joel S Burma, John R. Best, Caterina Rosano, Teresa Liu‐Ambrose, Cindy K Barha

**Affiliations:** ^1^ University of Calgary, Calgary, AB, Canada; ^2^ Simon Fraser University, Vancouver, BC, Canada; ^3^ University of Pittsburgh, Pittsburgh, PA, USA; ^4^ University of British Columbia, Vancouver, BC, Canada

## Abstract

**Background:**

The apolipoprotein E (APOE) ε4 allele is a well‐established genetic risk factor for cognitive decline and late‐onset Alzheimer's disease with stronger effects in females, while the ε2 allele may offer protective effects. Physical activity is known to mitigate cognitive decline; whether the degree of benefit differs by APOE genotype, sex, and race remains unknown.

**Methods:**

Analyses utilized data from 2,984 participants in the Health, Aging, and Body Composition cohort, comprising community‐dwelling black and white older adults followed for 10 years. Cognitive performance was assessed longitudinally using the Digit Symbol Substitution Test (DSST) for executive functions/processing speed and the Modified Mini‐Mental State Examination (3MS) for global cognition. APOE genotypes were categorized into ε2, ε3, and ε4 groups. Physical activity was quantified using self‐reported annual walking time. Linear models and latent growth curve modeling examined the interactions between APOE genotype, sex, race, and walking on cognitive trajectories with adjustments for study location, health score, age, education, and body mass index.

**Results:**

APOE ε4 carriers demonstrated steeper declines in both DSST and 3MS scores compared to ε3 carriers, irrespective of sex and race (βs>‐0.07, all *p* <0.034). There was some evidence that APOE ε2 was protective against cognitive decline, however associations were inconsistent across sex and race. Walking showed the strongest protective effect in ε4 carriers, slowing the decline in DSST among black females (β=0.39, *p* = 0.039), white females (β=0.60, *p* = 0.008), and white males (β=0.45, *p* = 0.009) (Figure 1). For 3MS, walking was associated with slower decline across ε4 carriers with slightly greater associations in black individuals (βs>1.11, *p* <0.001) compared to white (βs>0.67, *p* <0.031) (Figure 1). Walking was not associated with cognition decline in ε2 or ε3 carriers (Figure 1).

**Conclusions:**

APOE ε4 carriers displayed an elevated risk of cognitive decline; greater physical activity was significantly protective in this at‐risk group. Greater walking was associated with better executive function/processing speed in white ε4 carriers, and better global cognition in black ε4 carrier. These findings underscore the importance of tailoring public messaging about physical activity strategies to at‐risk vulnerable populations and highlight the intersection of genetic, sex, demographic, and lifestyle factors in cognitive aging research.